# Sensitivity of Four Various Candida Species to Photodynamic Therapy Mediated by Indocyanine Green, an in vitro Study

**DOI:** 10.30476/DENTJODS.2020.81817.0

**Published:** 2021-06

**Authors:** Atefeh Tavangar, Faezeh Khozeimeh, Mehdi Razzaghi-Abyaneh, Safieh Sherkat

**Affiliations:** 1 Dental Research Center, Dept. of Oral Medicine, Dental Research Institute, Isfahan University of Medical Sciences, Isfahan, Iran; 2 Dept. of Mycology, Pasteur Institute of Iran, Tehran, Iran; 3 Dept. of Oral Medicine of Shahrekord University of Medical Sciences, Shahrekord, Iran

**Keywords:** Candida, Indocyanine green, Laser, Nystatin, Photodynamic therapy, Photosensitizer

## Abstract

**Statement of the Problem:**

Various species of candida contribute to oral candidiasis. It is the time to shift from conventional rigid antimicrobial therapies to more patient specific and safer ones.

**Purpose:**

The present study aimed to investigate antifungal effects of photodynamic therapy (PDT) using Indocyanine green as photosensitizer and low-power laser irradiation on the viability of *candida albicans*, *candida tropicalis*, *candida glabrata* and *candida krusei*, and to compare it with Nystatin as the conventional treatment.

**Materials and Method:**

In this *in vitro* study, 0.5 McFarland suspensions of candida's species were prepared (n=50, each). Each strain was then divided into five groups of 10 samples each, according to the following experimental interventions: (1) Nystatin, (2) photodynamic therapy: laser irradiation (wavelength= 808 nm, power= 100 mW, energy density= 10 J/cm^2^, exposure duration= 100 s) in the presence of the photosensitizer, (3) laser irradiation alone, (4) treatment with the PS alone and (5-control: no exposure to laser light or photosensitizer. Next, serial dilutions were prepared and seeded onto Sabouraud dextrose agar. The colonies were counted, and the values of log (CFU/ml) were analyzed by variance and the Tamhan test (*p*< 0.05).

**Results:**

Photodynamic therapy mediated indocyanine green is significantly effective in reducing the number of CFU/ml of all species of candida tested, compared to control group (*p*< .001). Nystatin, laser irradiation and photodynamic therapy, with respectively decreasing potency, have considerably reduced the number of candida's colonies in all four bacterial strains (*p*<. 05). Photosensitizer alone, showed impressive antimicrobial potency against all species of candida except *candida albicans*, in comparison to control group.

**Conclusion:**

It seems that laser therapy alone is more powerful than photodynamic therapy mediated indocyanine green; however, conventional treatment has still the top antimicrobial efficacy towards all candida species.

## Introduction

Oral candidiasis is an opportunistic infection in the oral cavity. *Candida albicans* (*C.albicans*) is the most prevalent cause of oral mucosa candidiasis, which reaches to 60-70 percent of cases [ 1]. *C.albicans* the form of “non-pathogen," role as the normal flora of mouth, though under special conditions, it may lead to candidiasis [ 2]. Today, there are various antifungal medications introduced in this regard such as ionophores (Nystatin and Amphotericin B) and azole drugs. However, there are still some limitations in facile prescription of these drugs such as the bitter taste of Nystatin, which leads to nausea and patient intolerance [ 3], or the occurrence of drug resistance, especially to azoles, as was described in 81 percent of patients with HIV infection, who were under oral *C.albicans* therapy [ 4]. Moreover, oral candidiasis may occur iatrogenic as an adverse effect of other treatments such as chemotherapy for cancer suppression; in these patients, drug resistance was also abundantly observed [ 5]. These findings and coinfection with microbial flora such as *Pseudomonas Aeruginosa* necessitate a powerful, universal attempt to develop a novel strategy to remove fungal infections, without causing harmful effects or inducing resistance reactions [ 6- 8].

Photodynamic therapy (PDT) assisted laser is a new modality, based on non-toxic photosensitizer (PS) and safe light source. The combination of these items can trigger a biological cascade in the presence of radical oxygen for apoptosis and annihilation of microorganisms as long as malignant cells [ 9]. Different PSs (such as methylene blue, indocyanine green and toluidine blue) as well as diverse laser parameters were examined in this regard to achieve the best result in elimination of microbes and malignant cells. The indocyanine green as a new PS which followed by laser illumination, has shown promising effects against periodontal and peri-implant pathogens and malignant cells of melanoma [ 10- 12]. The present study was to compare the susceptibility of four various species of candida to PDT, PS, laser irradiation, and to conventional treatment of Nystatin.

## Materials and Method

### Study design

This in vitro investigation was employed for a total of 200 samples in four main categories of reference strains of candida species. Including *C.albicans* ATCC 10231, *Candida tropicalis* (*C.tropicalis*) PFCC 89-1456, *Candida glabrata* (*C.glabrata*) ATCC 90030 and *Candida krusei* (*C.krusei*) *DSM* 70079; 50 samples each. Four treatment modalities (including PDT, PS only, laser only and Nystatin) were examined on each sub-category of any strain of candida (N=10). Additionally, 10 samples in each group were selected randomly as control (N= 10), which did not receive any treatment or intervention.

### Microorganisms and culture conditions

Freeze-dried candida species have been subjected to revive the process. In order to prepare.5 McFarland, turbidity standard, the isolates were passage two times on Sabouraud dextrose agar plates 24 hours before providing the suspension of candida. Suspensions of candida's strains containing 10 (6) cells/ml were standardized in a spectrophotometer in530 nm wavelength (Biochrom WPA Lightwave II UV, Cambridge, UK).

In order to prevent microbial contamination and the irradiation of unwanted environmental lights, all steps were performed under a darkened biosafety hood at 28-degree centigrade temperature.

### Nystatin application

0.1 mL Nystatin (Jaber Ebne Hayyan Pharmaceutical Company, Tehran, Iran) (100,000 units per ml) was added to 0.1 ml suspensions of candida in microplates.

### Photodynamic therapy

PS application: Solutions of 5mg/ml of indocyanine green were used according to the manufacturer's manual (Diagnostic Green GmbH, Germany). Then, 0.1 ml of this solution was added to micro plates containing 0.1 ml suspensions of candida. 

Laser illumination: Laser was illuminated to micro plates containing a homogenized solution of 0.1 ml sterile saline and 0.1 ml candida. Laser parameters are summarized in Table 1.

**Table1:** Laser parameters and specifications

Parameter	Amount
Laser power	100 mW
WRadiation duration	100 sec
Dose	10 J/cm^2^
wavelength	808 nm
Probe status	No contact
Radiation mode	Continuous
Probecross section diameter	1 cm
Model	Polaris 2/SW PM2-25/M1/AP ver 4/0, (Star company, Bielsko-Biata, Poland)

PDT: Laser was illuminated to micro plates containing a homogenized solution of 0.1 ml Indocyanine green (Sigma, New York, York, USA) and 0.1 ml candida with the same parameters illustrated for laser treatment.

The mixing process of candida suspension with saline, PS or Nystatin was done on a shaker (Behdad company, Tehran, Iran), for 5min to achieve a well-homogenized solution. Eventually, using pour plate method 0.1 ml of each sample was added to 25ml Sabouraud dextrose agar medium, and then incubated at 37°C for 48 hours. Serial dilutions were then made from the samples contained in the tubes, and plated in duplicate onto Sabouraud dextrose agar culture medium. 

After 48 hours of incubation at 37°C, the viable colonies were counted and the values of CFU/ml were determined by colony count set (HYC-560 Digital Colony Counter, Hanyang Scientific Equipment Company-HYSC, Korea) (FFigure 1).

**Figure 1:**
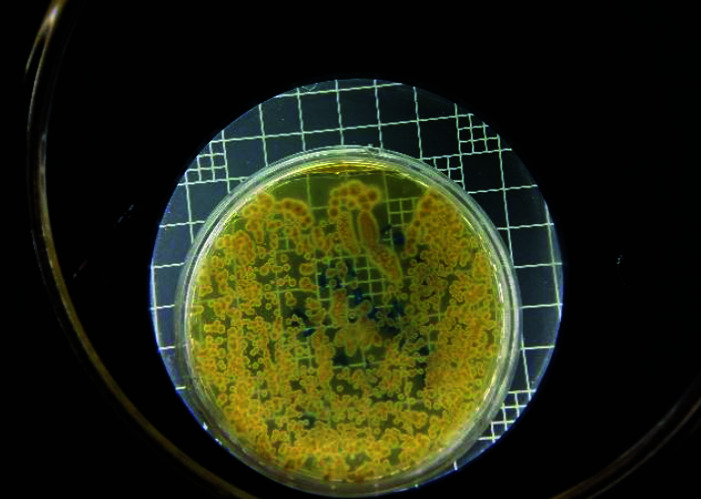
Colony count set

### Data analysis

The data have been statistically analyzed by SPSS23 software One-way ANOVA test and Tamhan test have been used to detect any significant difference between groups (*p*< 0.05).

## Results

*C.albicans* counts (CFU/ml) were transformed into base -10 logarithms. Therefore, one-way ANOVA with followed Tamhan test for multiple comparisons was performed (Table 2). The results of dual comparison of the study groups for each candida by "*p* Value”, are summarized in Table 3.

**Table2:** The number of candida colonies according to candida type methods treatment

		Interval of 95 percent reliability for average					
Candida strain	Group	Average	Standard deviation	Down limit	Up Limit	Minimum	Maximum
*C.albicans*	Control	1064.80	133.042	969.63	1159.97	910	1300
	PS	877.40	146.973	772.26	982.54	620	1094
	PDT	719.80	96.957	650.44	789.16	600	880
	Laser	614.60	89.399	555.66	673.54	530	730
	Nystatin	43.70	11.738	35.30	52.10	25	64
*C.tropicalis*	Control	925.30	74.005	872.36	978.24	829	1042
	PS	810.90	46.615	777.55	844.25	722	864
	PDT	706.50	49.718	670.93	742.07	640	780
	Laser	505.50	73.686	452.79	558.21	410	620
	Nystatin	85.00	19.872	70.78	99.22	48	116
*C.krusei*	Control	1279.40	98.067	1209.25	1349.55	1100	1432
	PS	922.50	70.352	872.17	972.83	800	1000
	PDT	742.10	102.499	677.78	814.42	600	949
	Laser	533.60	87.297	471.15	596.05	414	657
	Nystatin	218.40	35.211	193.21	243.59	176	276
*C.glabrata*	Control	1100.20	137.056	1002.16	1198.24	900	1300
	PS	846.40	105.833	770.69	922.11	730	1000
	PDT	675.30	104.368	600.64	749.96	514	810
	Laser	659.40	62.717	614.54	704.26	575	749
	Nystatin	0	0	0	0	0	0

**Table3:** The results of dual comparison of the study groups for each candida by "*p* Value"

Candida type	*p* Value	Comparison Group
*C.albicans*	0.088	Control- PS
	0.0	Control- PDT
	0.0	Control- Laser
	0.0	Control- Nystatin
	0.130	PDT- PS
	0.001	Laser-PS
	0.0	Nystatin-PS
	0.151	PDT- Laser
	0.0	PDT- Nystatin
	0.0	Nystatin- Laser
*C.tropicalis*	0.006	Control- PS
	0.0	Control- PDT
	0.0	Control- Laser
	0.0	Control- Nystatin
	0.002	PDT- PS
	0.0	Laser-PS
	0.0	Nystatin-PS
	0.0	PDT- Laser
	0.0	PDT- Nystatin
	0.0	Nystatin- Laser
*C.krusei*	0.0	Control- PS
	0.0	Control- PDT
	0.0	Control- Laser
	0.0	Control- Nystatin
	0.004	PDT- PS
	0.0	Laser-PS
	0.0	Nystatin-PS
	0.0	PDT- Laser
	0.0	PDT- Nystatin
	0.0	Nystatin- Laser
*C.glabrata*	0.001	Control- PS
	0.012	Control- PDT
	0.001	Control- Laser
	0.0	PS- PDT
	0.0	PS- Laser
	1.000	PDT- Laser

All interventions showed anti-fungal efficacy when compared to control, although species of *C.albicans* did not reduce effectively in comparison to control group when subjected to PS alone. In total, the highest number of candida's colonies was seen in a control group, while the least was achieved in Nystatin group. 

*C.glabrata*, which showed the most sensitivity to Nystatin, was eliminated. *C.krusei* showed the least sensitivity to Nystatin. Additionally, this trend was also found with PDT employment. C.tropicalis was recognized as the most sensitive strain to laser irradiation; inversely, *C.glabrata* was the most resistant to this. Surprisingly, PS only, also showed anti-fungal efficacy with the most promising effects on *C.tropicalis* and the least on *C.glabrata* strain.

Laser irradiation was significantly more effective than PDT against *C.krusei* and *C.tropicalis*; however, when the laser employed on *C.glabrata* and *C.albicans* strains, it showed a statistically equal anti-fungal power. 

PDT reduced candida colonies more effective than PS alone except in samples of *C.albicans*, which showed no statistically significant difference (*p*= 0.13).

## Discussion

Present investigation evaluated anti-fungal efficacy of PDT mediated indocyanine green on four common strains of candida. The number of colonies in all candida groups decreased significantly when subjected to PDT. In spite of the vast number of researches on the efficacy of PDT mediated indocyanine green, there are just two papers available about the effects of this modality on *C.albicans*. In addition, studies on the comparison of susceptibility of different species of candida to introduced remedies are scarce.

In an *in vitro* study performed by Fekrazad *et al*. [ 13], the effects of PDT mediated indocyanine green and new methylene blue against *C.albicans* was compared with control (no treatment). Similar to the results of present study, they reported a promising anti-fungal effect of PDT [ 13].

Azizi *et al*. [ 14], also investigated into the *in vitro* effects of PDT induced by indocyanine green and methylene blue on *C.albicans* and like the present study, compared this method with Nystatin; moreover, they used different laser parameters in laser dose and mode of radiation (pulse or continuous). Overall, in compliance with the current study results, authors reported a significant difference between the two modalities compared to control and as well between the two, Nystatin showed better results in *C.albicans* eradication than PDT. However, unlike our consequences, the anti-fungal efficacy of Nystatin was statistically equivalent to PDT, when pulse mode laser irradiation was induced by indocyanine green. 

Different PSs accompanying light illumination have been examined previously, in literature, as the novel anti- candida modulation such as methylene blue, toluidine blue, 5-aminolevulinic acid, and photofrin. Although there is not a univalent consensus on the results; one theory is nearly accepted by all, which defines that regardless of the type of PS, this modality can be used as an auxiliary treatment to conventional drug therapy [ 15- 18].

The type and concentration of PS, method of laser radiation, laser distance, laser dosimeter, and physiologic status of the aim microorganism, are all effective in the results of PDT by laser [ 19]. PS alone, in spite of having a light efficacy in candida annihilation has shown the least anti-fungal effects in the present and previous investigations compared to other treatment modalities [ 13- 14]. This conclusion demonstrated that the laser illumination following PS application in PDT is of paramount importance; therefore, it is suggested to make forthcoming investigations with a focus more on selecting the best laser parameters' layout than the type or characteristics of PS.

The characteristics of the aimed microorganism have a key role in PDT success rate. *C.albicans* seems to be more resistant than gram-positive bacteria against this treatment modality. It is suggested that perhaps the presence of nuclear membrane, larger cell size, and the fewer target areas for free oxygen radicals per unit of cell volume in *C.albicans* may play a significant role in this resistance [ 20]. In the present study as well, *C.albicans* was more resistant to PDT compared to other employed species of candida. 

Maximum absorption of light by colored PS molecules is also an important issue when applying PDT. The wavelength of radiation must be set where there is the most absorbance by the PS molecules. This will produce a maximum amount of oxygen free radical to eliminate the target microorganism. For example, the most absorbent of the indocyanine green is in the range of 805-810 nm [ 21- 22]. Therefore, it seems that the wavelength used in the present study was optimum; however, the authors believe that higher-energy density of the laser was needed to achieve better results. Unfortunately, in previously published studies, different laser parameters with different conditions and methods have been examined, which make the comparison and consensus between various and contradictory results very difficult [ 14, 23]. In the present study laser, the parameters were chosen according to Azizi *et al*. [ 14] investigation. 

One of the critical concerns about laser application on vital tissues is the laser side effect in increasing the targeted tissue temperature, shifting the tissue healing pathways to unwanted and harmful ones. Nonetheless, it is demonstrated that diode lasers in comparison to other laser types would produce less heat [ 24]. Silva *et al*. [ 25] reported an increase of 2 centigrade in temperature of target tissue when applying PDT (by diode laser) for 30, 60, and 120 seconds, which can be negligible. In another study conducted by Hirata et al. [ 26], laser application in power ranging 50-500mW for 2min duration caused moderator effects on mammalian cell proliferation *in vitro* [ 26]. It seems that these side effects could be less prominent when time duration of laser illumination is reduced. The toxicity of indocyanine green has not been demonstrated, up to date [ 25].

Wainwright *et al*. [ 27] demonstrated that microorganism's eradication by PDT depends on the chemical formula of PS and duration of drug exposure to bacterial cells. The cell membrane acts as a selective barrier against PS diffusion; therefore, the PS’s penetration into cells would be done through an active transfer mechanism. Damaging the cell membrane of bacteria, increasing the permeability of the cytoplasmic membrane, and intervention with DNA replication are probable mentioned mechanisms of action of PDT [ 27].

Previous data confirmed PDT usability in dental practice for anti-microbial purposes [ 28- 29]. The effects of PDT induced by indocyanine green have been demonstrated as an effective treatment modality for periodontal disease [ 11- 12] and also for eradication of melanomas and acne vulgaris [ 10, 30]. Nevertheless, there are still some unknown points, which prevent it to be as a standard modality; it seems that *in vitro* studies are yet needed to standardize various options and parameters introduced in literature. Furthermore, comparative investigations with conventional therapies should be held to assess the cost benefit of this novel modality. In the present study, Nystatin showed significant better results than PDT, so the authors of this study propose anti-candida PDT application when Nystatin therapy is impossible or tolerated or as a conjunctive therapy to traditional treatments. 

Indocyanine green is approved by Food and Drug Administration (FDA) and has been used for diagnostic purposes in the field of medicine, such as detection of capillary roots [ 31]. Absorption wavelength of indocyanine green is at 805 nm. It is demonstrated that indocyanine green binds to plasma proteins, so does not lead to chemical changes in the body [ 11].

In the present study, the efficacy of laser with or without PS, application was nearly equal, while Fekrazad *et al*.’s [ 13] investigation of laser illumination following indocyanine green application showed considerable better anti-fungal results than laser alone. This may be due to different brands of indocyanine green used in the two investigations, as we used Cardiogreen and they employed Emundo (both by Sigma Aldrich Company). Accordingly, it seems that Emundo has more potency in this field of application than Cardiogreen. Nonetheless, this hypothesis should be examined more specifically, in a matched comparative study. Moreover, laser parameters and mode of PS application as well as the method of PS dilution were different in these two investigations; each one could have a critical role in achieving dissimilar results.

## Conclusion

Under the conditions of the present study, susceptibility of all species of candida was primarily to Nystatin. PDT is significantly effective in reducing the candida colonies. PS alone, showed sufficient antimicrobial potency against all species of candida except *C.albicans*, in comparison to control group. The outlook for new treatments in candidiasis seems to be very clear. Further studies on the efficacy of these treatments are recommended.
